# The Cancer Exome Generated by Alternative mRNA Splicing Dilutes Predicted HLA Class I Epitope Density

**DOI:** 10.1371/journal.pone.0038670

**Published:** 2012-09-25

**Authors:** Thomas Stranzl, Mette V. Larsen, Ole Lund, Morten Nielsen, Søren Brunak

**Affiliations:** Center for Biological Sequence Analysis, Department of Systems Biology, Technical University of Denmark, Lyngby, Denmark; University of Oslo, Norway

## Abstract

Several studies have shown that cancers actively regulate alternative splicing. Altered splicing mechanisms in cancer lead to cancer-specific transcripts different from the pool of transcripts occurring only in healthy tissue. At the same time, altered presentation of HLA class I epitopes is frequently observed in various types of cancer. Down-regulation of genes related to HLA class I antigen processing has been observed in several cancer types, leading to fewer HLA class I antigens on the cell surface. Here, we use a peptidome wide analysis of predicted alternative splice forms, based on a publicly available database, to show that peptides over-represented in cancer splice variants comprise significantly fewer predicted HLA class I epitopes compared to peptides from normal transcripts. Peptides over-represented in cancer transcripts are in the case of the three most common HLA class I supertype representatives consistently found to contain fewer predicted epitopes compared to normal tissue. We observed a significant difference in amino acid composition between protein sequences associated with normal versus cancer tissue, as transcripts found in cancer are enriched with hydrophilic amino acids. This variation contributes to the observed significant lower likelihood of cancer-specific peptides to be predicted epitopes compared to peptides found in normal tissue.

## Introduction

Cancer-specific splice variants are of significant interest as they may be involved in pathogenesis and may further potentially be used as biomarkers and generate novel targets for cancer [1], [2]. The human immune system is capable of responding to some of these cancer specific antigens, as first shown by a melanoma-specific antigen, MAGE-1, able to stimulate human T cells [3], [4]. More generally, individuals with high or medium cytotoxic activity of peripheral-blood lymphocytes are further associated with a significantly lower risk of cancer, suggesting a role for natural immunological host defense mechanisms in cancer [5].

Alternative splicing can change the structure of mRNA by inclusion or skipping of exons, and this may alter the function, stability or binding properties of encoded proteins and thereby contribute to human diseases such as cancer [6]. In a study investigating alternative splicing events in ovarian and breast tissues affected by tumors it was found that about half of all splicing events in these tissues are altered in tumors, many of them due to exon skipping [7]. Similar trends have been observed in other types of cancers, e.g., in colon cancer and testicular tumor [8], [9], as well as in gastric cancer, where genes showing differential expression between cancer cell lines and corresponding normal tissues were found [10]. In addition to cancer being involved in dysregulating pathways, thus contributing to changes in alternative splicing and gene expression controlled by these proteins [11], human leukocyte antigen (HLA) class I antigen processing components and HLA expression have also been shown to be downregulated in connection with cancer [12], [13]. A study investigating alterations of HLA class I expression in 12 ovarian cancer patients reported low levels of HLA class I antigens in tumor cells from all patients. One patient-derived tumor cell line showed a complete haplotype loss, including the HLA-A2 locus [14].

These observations are interpreted as mechanisms adopted by tumors to escape immune surveillance and to avoid tumor cell recognition and destruction [15], [16]. It has been suggested that elimination of growing tumors by the immune system may lead to selection of tumor variants that are efficient in avoiding immune system recognition [17]. There thus seems to be accumulative evidence for cancer being coupled to alternative splicing as well as to an efficacy in evasion from the immune system by downregulation and altering HLA expression. Most of the studies relating cancer-specific alternative splicing to altered immune system surveillance are, however, of limited size and in most cases anecdotal. Here, we wanted to investigate, in a large-scale study, if the alternative cancer exome already at the step of mRNA splicing contains a bias compared to normal transcripts in the set of possible HLA class I epitopes.

## Results

### Transcripts over-represented in cancer contain fewer predicted epitopes restricted by the three most common HLA class I supertypes

The aim of this study was to investigate, using a large-scale data set, if peptidomes specific for cancer versus normal tissue have different properties related to altered degree of immune system surveillance. To do this, we constructed two sets of peptides, one over-represented by cancer tissue and one over-represented by normal tissue. Globally permutated versions of these sets were produced as described in Material and Methods. The global permutation destroys structural characteristics within the HLA-binding 9-mers, only maintaining global compositional properties. For comparison, we constructed locally permutated normal and cancer sets by permutating each peptide separately, thus preserving the local amino acid composition of each peptide. To investigate immune-related properties, potential epitopes covering all 12 HLA class I supertypes were predicted using NetMHCpan. For each supertype, we calculated the percentages of predicted epitopes for the six peptide data sets: normal, normal globally and normal locally permutated, cancer and cancer globally and cancer locally permutated.

It is well known that some HLA class I supertype representatives are more common than others. It is therefore expected that for the less frequent HLA alleles, the results are more likely to include noise. The source of our data set, the ASTD database, is to a large extent originating from EST data without HLA specific information. EST data is mostly based on Caucasian Europeans [18]; therefore we can safely assume that the more common HLA types in the European population are also more common in our dataset. The HLA allele frequencies were obtained from the dbMHC database [19]. Approximate numbers of expected phenotype per supertype in the European population are given in Table 1.

**Table 1:** Phenotype frequencies.

Allele	Frequency
HLA-A*02:01	0.47
HLA-A*01:01	0.30
HLA-A*03:01	0.26
HLA-B*07:02	0.24
HLA-B*08:01	0.22
HLA-A*24:02	0.13
HLA-B*40:01	0.10
HLA-B*15:01	0.07
HLA-B*27:05	0.06
HLA-A*26:01	0.05
HLA-B*39:01	0.02
HLA-B*58:01	0.02

HLA frequencies in the European population. Data obtained from the dbMHC database [19].

**Figure 1:**
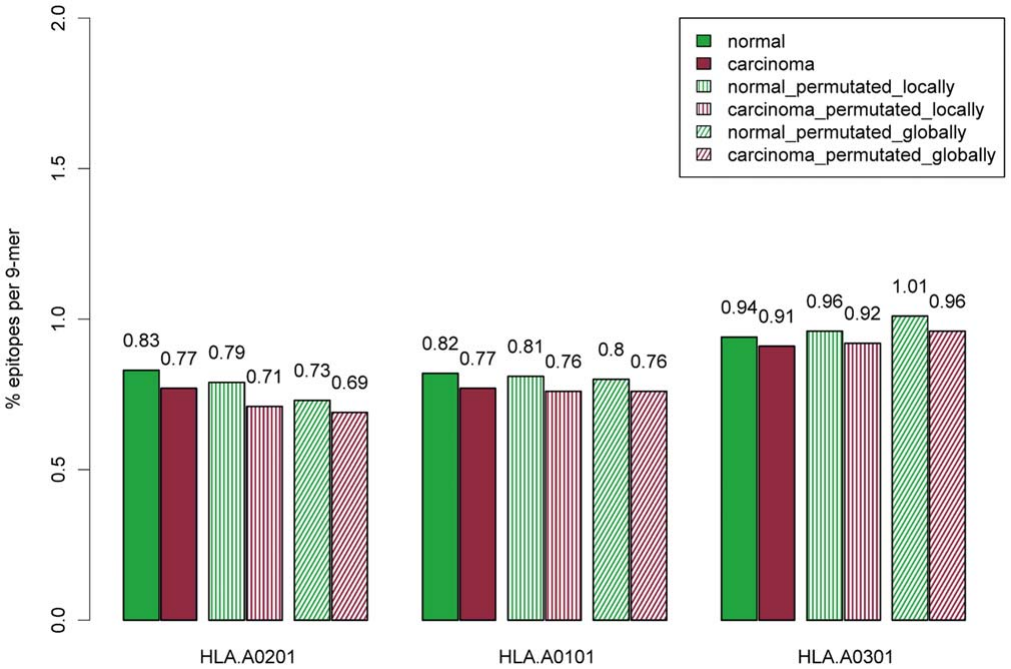
Percentage of epitopes per 9-mer comparison. Data is shown for the three most common HLA-I alleles in the European population. Each bar shows the percentage of predicted epitopes per 9-mer in the respective set. Each set consists of peptides that are either over-represented in normal or cancer tissue. Globally permutated or locally permutated version of the peptide sets were constructed as described in Materials and Methods. All observed differences between cancer and normal tissues are significant (p<0.006, 2-sample test for equality of proportions).

The three most common supertype representatives in the European population are HLA-A*02∶01, HLA-A*01∶01 and HLA-A*03∶01. For these three supertype representatives, the transcripts associated with normal tissue have a significantly higher percentage of predicted epitopes than transcripts over-represented in cancer. Figure 1 shows the observed numbers, in percentages of predicted epitopes per 9-mers, for the different data sets for these three most common supertype representatives. All observed differences between normal and cancer tissues shown in Figure 1 are significant (p<0.006, 2-sample test for equality of proportions).

### For most HLA class I supertypes, cancer transcripts contain fewer predicted epitopes

Further, the percentage of predicted epitopes for permutated and not-permutated sequences for all 12 supertype representatives is shown in Table 2. Here, we observed a similar tendency as compared to our observation for the three most common supertypes in the European population. For non-permutated sequences, seven out of the twelve supertype representatives (HLA-A*01∶01, HLA-A*02∶01, HLA-A*03∶01, HLA-A*24∶02, HLA-A*26∶01, HLA-B*15∶01 and HLA-B*58∶01) had a significant lower fraction of predicted epitopes in sequences assigned to cancer pathology. A statistical significant difference, where cancer-associated peptides contained more predicted epitopes was, on the other hand, only observed for one supertype representative, namely HLA-B*27∶05.

**Table 2:** Epitopes per set for all supertype representatives.

	% epitopes				% epitopes globally permutated				% epitopes locally permutated			
Allele	N	C	N/C	P-val	N	C	N/C	P-val	N	C	N/C	P-val
HLA-A*01:01	0.82	0.77	1.06	0.000	0.80	0.76	1.06	0.000	0.81	0.76	1.07	0.000
HLA-A*02:01	0.83	0.77	1.08	0.000	0.73	0.69	1.05	0.000	0.79	0.71	1.10	0.000
HLA-A*03:01	0.94	0.91	1.04	0.002	1.01	0.96	1.05	0.000	0.96	0.92	1.04	0.005
HLA-A*24:02	0.89	0.79	1.13	0.000	0.77	0.70	1.11	0.000	0.89	0.77	1.15	0.000
HLA-A*26:01	0.76	0.71	1.07	0.000	0.70	0.66	1.06	0.000	0.71	0.68	1.05	0.001
HLA-B*07:02	1.29	1.30	1.00	1.000	1.27	1.29	0.99	1.000	1.25	1.28	0.97	0.054
HLA-B*08:01	1.02	1.03	0.99	1.000	1.00	0.99	1.01	1.000	0.97	0.97	1.00	1.000
HLA-B*15:01	0.86	0.79	1.09	0.000	0.83	0.77	1.08	0.000	0.85	0.79	1.07	0.000
HLA-B*27:05	0.99	1.02	0.97	0.021	0.99	1.00	0.98	1.000	1.04	1.04	0.99	1.000
HLA-B*39:01	0.97	0.96	1.02	0.985	1.05	1.02	1.02	0.221	1.01	1.00	1.01	1.000
HLA-B*40:01	0.87	0.89	0.98	1.000	1.03	1.06	0.98	0.325	0.95	0.99	0.96	0.002
HLA-B*58:01	1.01	0.91	1.11	0.000	0.99	0.89	1.10	0.000	0.98	0.91	1.08	0.000

Percentage of predicted epitopes is given for data extracted from the ASTD database as well as for permutated sequences. N/C is the ratio between the normal and cancer percentages. P-values are calculated by two-tailed t-test and adjusted for multiple testing by Bonferroni correction.

When analyzing permutated sequences, similar results were observed. Only one supertype representative (HLA-B*40∶01, locally permutated) had significantly more predicted epitopes in the permutated cancer sequences than in the permutated normal sequences. On the other hand, permutated, normal sequences had consistently for both the local and global permutated sets more predicted epitopes for seven supertype representatives (HLA-A*01∶01, HLA-A*02∶01, HLA-A*03∶01, HLA-A*24∶02, HLA-A*26∶01, HLA-B*15∶01, HLA-B*58∶01). For these seven supertype representatives, the difference between normal and cancer data sets is significant in the permutated as well the non-permutated data sets. The observation that cancer transcripts contain fewer predicted epitopes for most HLA class I supertype representatives, is stable, when different thresholds for the prediction of potential epitopes are applied (data not shown).

### HLA motif and amino acid composition biases

The relative difference in predicted epitope density between normal and cancer is, for our previously defined most common HLA alleles, relatively stable. Also, the difference in epitope density is largest when comparing non-permutated to globally permutated peptide sets. For HLA-A*02∶01, a noticeable decrease of predicted epitopes is observed when comparing normal and cancer non-permutated peptides to normal and cancer permutated peptides. As seen from Table 2 and Figure 1, the difference in percentage of epitopes is the largest when comparing the non-permutated sequences to the globally permutated sequences (normal: 0.83 vs 0.73, cancer: 0.77 vs 0.69). For HLA-A*01∶01, the percentage of epitopes in non-permutated versus permutated sequences appears to be relative stable (normal: 0.82 vs 0.80, cancer: 0.77 vs 0.76), whereas permutated HLA-A*03∶01 sequences have more predicted epitopes than the corresponding non-permutated sequences (normal: 0.94 vs 1.01, cancer: 0.91 vs 0.96). For these three supertype representatives, the percentage of predicted epitopes in locally permutated peptides always falls between the respective percentages for non-permutated and globally permutated sequences. Locally permutated peptides preserve only local amino acid composition, and globally permutated peptides have their local structural properties destroyed and preserve only global amino acid composition. These observations indicate that both global and local structural amino acid properties are factors that define the observed differences in the epitope densities between the normal and cancer peptidome.

**Figure 2:**
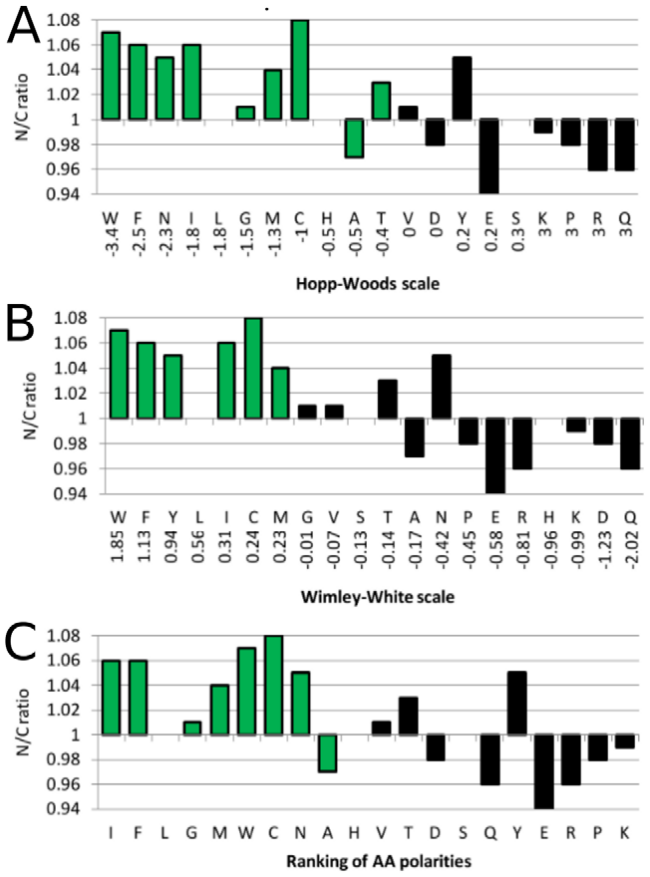
Hydrophilic amino acids are enriched in cancer. N/C ratios in relation to Hopp-Woods hydrophilicity scale (A), Wimley-White hydrophobicity scale (B) and to the mean ranking of amino acids based on 38 hydrophobicity scales (C). N/C ratio is the ratio of observed frequencies of the respective amino acids in polypeptides of over-represented transcripts from normal and cancer tissues. If the N/C value >1, the amino acid is more common in normal tissue; If the N/C value <1, the amino acid is more common in cancer. Green bars refer to more hydrophobic amino acids, whereas black bars refer to more hydrophilic amino acids. All N/C ratios larger or smaller than 1 are significant (p<0.001, calculated using the Wilson score [45] and Bonferroni corrected).

An analysis of relative amino acid composition was performed for all over-represented 9-mers associated with normal and cancer. We found that hydrophilic residues are more common in polypeptides from cancer transcripts than from the normal polypeptides. The relations of N/C ratios compared to the hydrophilicity scale of amino acids by Hopp-Woods, the hydrophobicity scale by Wimley-White as well as the mean ranking of amino acids according to the frequency of their occurrence for 38 published hydrophobicity scales are shown in Figure 2. In Figure 2, residues are more common in cancer if N/C is smaller than 1. Hydrophilic residues are marked black.

The Hopp-Woods and Wimley-White scales correlate strongly with the N/C ratios with a Spearman rank correlation coefficient of −0.72 and 0.78, respectively. The mean ranking amino acid scale is correlated with a correlation coefficient of −0.65. All three correlation coefficients are significant (P-value <0.003, exact permutation test). No correlation was found for other amino acid properties like mass, surface area or volume (data not shown).

It is striking to observe that all strong hydrophilic amino acids (KPRQ, Hopp-Woods scale) are enriched in sequences associated with cancer. A similar observation is made for Wimley-White scale: We identified seven amino acids significantly more common in cancer (APERKDQ). Six of these (all except A) are within the seven most hydrophilic amino acids based on the Wimley-White scale. A reversed trend is found for hydrophobic amino acids. The top significant amino acids classified by both Hopp-Woods and Wimley-Scott as hydrophobic (WFICM) are all more common in sequences associated with transcripts from normal tissue.

**Figure 3:**
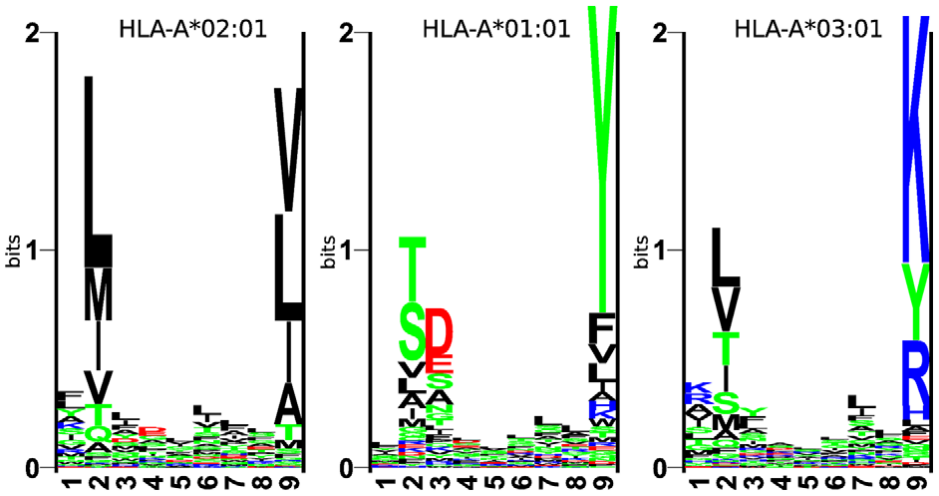
Human HLA motifs. The three most common HLA types in the European population. The height of a column of letters is equal to the information content at that position, whereas the height of each letter within a column is proportional to the frequency of the corresponding amino acid at that position [44].

Based on these findings, one could suggest an explanation for the difference in epitope density between the normal and cancer peptidome. The binding motifs for the 3 most frequent supertype representatives are shown in Figure 3. Out of the four most preferred amino acids at the HLA-A*02∶01 anchor positions, three amino acids (VMI) are enriched in normal transcripts, whereas only one (L) is as common in normal as in cancer. This leads to the conclusion that at least part of the observed differences in percentage of predicted epitopes in normal versus cancer transcripts are due to amino acid composition. The same tendency is found for HLA-A*01∶01. The two most frequent amino acids in the motif (YT) are also more often found in normal tissue, whereas S is neutral and the next common amino acid, D, is more common in cancer. The most frequent amino acid for HLA-A*03∶01(K) is slightly more common in cancer, whereas the second-next frequent (Y) is, due to a stronger preference to fit peptides from normal tissues, shifting the bias towards amino acids more common in splice variants associated with normal tissue. For all three motifs, we further calculated average weighted biases, based on N/C ratios and amino acid frequencies (see materials and methods). The weighted biases were calculated for both the respective 5 most frequent amino acids per motif as well as all 20 amino acids. For all three motifs we observed an overall preference for amino acids found in our normal tissue set.

## Discussion

Alternative splicing of mRNA transcripts is an important mechanism for generating genomic complexity and has been shown to differ between cancer and the corresponding normal tissues [1], [8], [9]. In addition, cancers in some cases downregulate HLA class I antigen-processing components and HLA class I expression to avoid detection by the immune system. These observations led us to investigate whether transcripts found in cancer tissue share characteristics that would reduce immune system recognition. Here, we have carried out a large-scale analysis aiming at identifying immune system related imprints that can differentiate cancer from normal transcripts. Based on ASTD database, a database providing predicted splice forms, we identified two peptide data sets; one associated with transcripts over-represented in cancer and one associated with transcripts over-represented in normal tissue. Using state-of-the-art immunoinformatics prediction tools, we next analyzed the two data sets for differences in terms of likelihood of being presented on prevalent HLA class I molecules, and hence potential for activating the immune system.

We found that peptides, which due to alternative splicing are expressed in cancer tissue, contain fewer predicted epitopes restricted by the three most common HLA class I alleles than peptides expressed in normal tissue. Using globally permuted data sets we consistently, for the three most common HLA class I alleles, found that the observed loss in epitope density in the cancer peptidome is maintained also for the permuted data sets. This strongly indicates that differences in amino acid composition between peptides from alternatively spliced normal and cancer transcripts are the driving force of the reduced predicted epitope density.

The reason for the observed change in frequency of specific amino acids in proteins associated with cancer as compared to normal tissue is unknown, but the phenomenon has previously been observed in studies aiming at identifying biomarkers for early stage detection of cancer: In a recent study, the levels of alanine, isoleucine, leucine and valanine were found to be increased in the pancreases of rats with pancreatic cancer as compared to samples from rats with chronic pancreatitis and healthy rats [20]. In another study, the levels of N-methylalanine and lysine were found to be significantly increased in the plasma from pancreatic cancer patients, while the level of glutamine and phenylalanine was found to be decreased [21]. These studies identified differences in amino acid composition in a single cancer type based on blood plasma and tissue samples. We, in contrast, analyze over-represented cancer peptides in general. As to be expected, the findings regarding amino acid concentration reported in this study are not concurrent with those of the single cancer type studies.

A possible explanation as to why we observed fewer predicted epitopes in peptides, which due to alternative splicing are expressed in cancer, could be that the host's immune system restricts the cancer exome. In that case, pressure from the immune system disfavors cancer cells that present new epitopes at the cell surface. An alternative explanation – which does not exclude the previous explanation – takes as starting point the observed change in amino acid frequency, especially the increase in hydrophilic amino acids in cancer proteins.

It has previously been shown that missense mutations in the BRCA domain of high-risk breast and ovarian cancer patients frequently target strongly hydrophobic amino acids [22]. Further, it has been suggested that the stabilization of a protein structure is to a large part due to the hydrophobic effect [23]. Accordingly, the increase in hydrophilic amino acids has a destabilizing effect on protein structure, which is in concordance with the protein loss-of-function that is correlated with cancer progression. This is exemplified by a study concerning inherited missense mutations of the tumor suppressor gene, *BRCA-1*, which may predispose to breast or ovarian cancer [24]. In this study, it was found that the mutations predominately target conserved hydrophobic amino acids that are responsible for folding and stability. Since, in particular, the most common HLA class I allele, A*02∶01, prefers hydrophobic amino acids at the anchor positions, an increase in hydrophilic amino acids will inevitably lead to fewer predicted epitopes. The reduction in epitope density in peptides associated with cancer might therefore be an intrinsic property of proteins that are destabilized by a decrease of hydrophobic amino acids as part of the progression to cancer. We are, however, not aware of studies that show a general increase of hydrophobic amino acids throughout different forms of cancer.

A bias of fewer potential epitopes due to cancer was previously shown by Wiedenfeld et al [25]. Peptides caused by missense mutations of p53 were shown to have a decreased fit to the HLA-A*02∶01 motif. All predicted variants of the peptides were either from patients with other alleles or the allele was lost during tumorigenesis. The decreased fit to the HLA motif due to mutations is in coherence with our study investigating differences in epitope density due to alternative splicing. To our knowledge, this is the first study indicating that alternatively spliced cancer transcripts tend to express fewer potential epitopes than alternatively spliced transcripts found in normal tissue. The identified difference in amino acid composition towards hydrophilic amino acids in the alternative spliced cancer exome is a possible explanation for the bias in potential HLA class I epitopes. The preference for hydrophilic amino acids at the step of alternative mRNA splicing could support the development of cancer by providing it with the possibility of evading the host's immune system. In this case by leading to fewer potential HLA class I epitopes presented at the cell surface.

## Materials and Methods

### Data extraction from the ASTD database

The Alternative Splicing and Transcript Diversity database (ASTD) provides access to a collection of alternative splice events and transcripts of genes from human, mouse and rat [26]. The aim of the database is to analyze the mechanisms of alternative splicing on a genome-wide scale. It integrates a computational pipeline for detection and characterization of isoform splice patterns as well as alternative introns and exons. The database contains predicted transcripts, generated by mapping expressed sequence tags (ESTs) to genome sequences. Our study is based on ASTD version v1.1 build 9 (accessible at ftp://ftp.ebi.ac.uk/pub/databases/astd/). The database covers 14,194 human genes and lists 50,581 unique transcripts not covered by Ensembl genes. Based on related evidences from cDNA libraries, many of these transcripts are tagged with pathology information. The pathology information is given as eVOC ontologies, which is a controlled vocabulary for unifying gene expression data [27]. As an alternative to the ASTD database, we would have liked to use RNA sequence data, but could not identify any usable database, providing genome-wide coverage of potential transcripts, together with pathology information.

Two data sets were generated based on annotated pathology information. All transcripts tagged with the information of being expressed in normal tissue were assigned to subset N. This subset consisted of 30,739 transcripts derived from 11,980 genes. A second subset, C, with transcripts related to cancer, consisted of 27,967 transcripts derived from 10,730 genes.

The cancer subset consists of all transcripts tagged with eVOC terms related to cancer; that is being a subgroup of tumor in the eVOC ontology hierarchy (Table 3). Several eVOC terms can be associated to the same transcript.

**Table 3:** eVOC terms used for cancer subset.

Burkitts lymphoma	Glioblastoma	Myeloid leukemia
Ewings sarcoma	Glioma	Myeloma
T-cell leukemia	Hypertrophic cardiomyopathy	Neoplasia
Wilms tumor	Insulinoma	Neuroblastoma
Adenocarcinoma	Leiomyosarcoma	Oligodendroglioma
Adenoma	Leukaemia	Osteosarcoma
Astrocytoma	Liposarcoma	Papillary serous carcinoma
Carcinoid	Lymphoblastic leukemia	Phaeochromocytoma
Carcinoma	Lymphocytic	Polyp
Carcinoma in situ	Lymphoma	Retinoblastoma
Chondrosarcoma	Aalignant tumour	Rhabdomyosarcoma
Choriocarcinoma	Medulloblastoma	Sarcoma
Enchondroma	Melanoma	Seminoma
Fibrosarcoma	Meningioma	Teratocarcinoma
Fibrothecoma	Monocytic leukemia	Tumour

For our analysis, we were interested in transcripts uniquely associated to normal tissue or to one or more of the cancer eVOC terms. Two new subsets consisting of transcripts only associated to either normal or cancer eVOC terms were created. Out of 30,739 transcripts associated to normal, 16,566 were uniquely associated with normal tissue, due to ASTD database, and not with cancer (unique N set). The subset of transcripts uniquely associated with cancer (unique C set) consists of 13,794 transcripts (see Table 4). Transcripts covered by each data set are unique for either normal tissue or cancer as defined by the ASTD database. The ASTD database does not provide pathology information for all transcripts nor lists all potential tissue types or pathologies. Accordingly, we refer to our sets of transcripts uniquely associated to either normal or cancer as over-represented in either normal or cancer tissue.

**Table 4:** Number of transcripts and genes per set.

	Normal	Cancer
Number of transcripts	30,739	27,967
Number of genes	11,980	10,730
Number of uniquely associated transcripts	16,566	13,794
Number of uniquely associated genes	8,741	7,128
Average number of unique transcripts/gene	1.90	1.94

Transcripts were extracted from the ASTD database. Number of transcripts and genes associated with normal and cancer pathology terms are given.

### Translation to proteins

All transcripts assigned to either normal or cancer pathology were translated to their respective protein sequence using Virtual Ribosome [28]. The longest ORF among all three reading frames was chosen as the translated protein sequence. The protein sequence and corresponding transcript were discarded if no ORF was found or if the resulting protein sequence was shorter than 9 amino acids. The threshold of 9 amino acids was chosen as we subsequently apply the epitope prediction on 9-meric peptides, although we are aware that proteins this small might not be functional. Applying this filter resulted in a normal set of 16,490 transcripts and a cancer set of 13,721 transcripts.

### Generation of unique 9-mers

All proteins assigned to either normal or cancer pathology states were divided into overlapping 9-meric peptide sequences. Peptide sequences that were found in both groups were removed, leading to the creation of two sets of unique 9-mer peptides. There are 1,856,231 unique 9-mers in the normal group (N-peptidome) and 1,684,028 unique 9-mers in the cancer group (C-peptidome). Note that normal and cancer sets do not consist of complete proteins; they only consist of unique 9-meric peptides not found in the other set. Permutated sets of both the unique N and unique C set were created. For each set, one locally permutated and one globally permutated set of 9-meric peptides was generated. The local permutated sets were constructed by permuting each 9-mer, thus keeping the amino acid composition within each 9-mer fixed. The global permutated sets were made by randomly constructing new 9-mers out of all amino acids within each set. This preserves the overall amino acid composition within the unique N and C sets, local properties within each 9-mer are, however, destroyed.

### Prediction of potential HLA class I epitopes

The prediction method NetMHCpan-2.4 [29], [30] was used for predicting potential epitopes for the 12 HLA class I supertypes [31]. The NetMHCpan-2.4 method was trained on an experimentally validated data set of more than 100,000 quantitative peptide – HLA class I interactions covering more than 100 HLA molecules and has been evaluated as the best pan-specific method for HLA peptide binding in a large benchmark study [32]. A general accepted threshold for binding is a rank score of 1% [33], [34] (binding strength falling within the top 1% compared to a large set of random natural peptides), which is also the threshold, used throughout this study.

The percentages of potential epitopes per 9-mer for all 6 sets (normal 9-mers, normal globally permutated 9-mers, normal locally permutated 9-mers, cancer 9-mers, cancer globally permutated 9-mers and cancer locally permutated 9-mers) were calculated. P-values for difference in percentage of predicted epitopes between normal and cancer 9-mers for non-permutated and permutated subsets were calculated by a 2-sample test for equality of proportions and adjusted for multiple testing (Bonferroni correction).

### Amino acid scales

The amino acid abundance for normal tissue compared to cancer tissue was determined based on all unique 9-mers in the two data sets. The relative frequencies for all amino acids in both the normal and cancer sets were calculated. Observed ratio of frequencies (N/C) of amino acids among normal and cancer tissues was correlated with Hopp-Woods hydrophilicity [35] and Wimley-White hydrophobicity scale [36] values. The ratio was further correlated with a mean ranking scale per amino acid as published by Simpso]. According to Simpson [37], the scale is based on the mean ranking of amino acids according to the frequency of their occurrence at each sequence rank for 38 published hydrophobicity scales [38]. Other investigated scales are average volume of buried residues [39], [40], van der Waals volume [41] and total accessible surface area [42].

Bootstrapping was applied to test if an amino acid property scale is correlated with enriched expression of residues in either unique normal or cancer 9-mers. For each scale, the Spearman rank correlation coefficient was calculated and the significance of the correlation was estimated using exact permutation test.

### HLA motif bias

HLA binding motifs were generated from NetMHCpan-2.4 training data. Position specific weight-matrices were calculated using sequence weighting and correction for low counts [43]. Sequence logos were visualized as described by Schneider and Stephens [44], where each letter represents its proportional frequency of the corresponding amino acid at that position. Based on amino acid frequencies and observed ratio of frequencies (N/C) of amino acids among normal and cancer tissues, we calculated for the HLA-A*A02∶01, HLA-A*A01∶01 and HLA-A*A03∶01 motifs their respective overall bias towards either our defined normal or cancer peptide set. This was done for all 20 amino acids and for the 5 most frequent amino acid occurrences per motif. Per position, the tendency to fit preferably to either the normal or the cancer peptidome was calculated by summation of the respective amino acid frequencies multiplied with the related N/C values for all 20 amino acids. Likewise the calculation for the 5 most frequent amino acid occurrences per motif, where only the subset of the motifs 5 most frequent amino acid occurrences at this position is considered. Similar to the N/C ratio, a motifs bias to preferably fit to our normal set is given, if the average over all position for a motif is larger than 1.

## References

[1] Thorsen K, Sørensen KD, Brems-Eskildsen AS, Modin C, Gaustadnes M, et al (2008) Alternative splicing in colon, bladder, and prostate cancer identified by exon array analysis. Molecular & cellular proteomics: MCP 7: 1214–1224. 18353764 10.1074/mcp.M700590-MCP200

[2] Skotheim RI, Nees M (2007) Alternative splicing in cancer: noise, functional, or systematic? The international journal of biochemistry & cell biology 39: 1432–1449. 17416541 10.1016/j.biocel.2007.02.016

[3] van der Bruggen P, Traversari C, Chomez P, Lurquin C, De Plaen E, et al (1991) A gene encoding an antigen recognized by cytolytic T lymphocytes on a human melanoma. Science (New York, NY) 254: 1643–1647. 10.1126/science.1840703 1840703

[4] Finn OJ (2008) Cancer immunology. The New England journal of medicine 358: 2704–2715. 18565863 10.1056/NEJMra072739

[5] Imai K, Matsuyama S, Miyake S, Suga K, Nakachi K (2000) Natural cytotoxic activity of peripheral-blood lymphocytes and cancer incidence: an 11-year follow-up study of a general population. Lancet 356: 1795–1799. 11117911 10.1016/S0140-6736(00)03231-1

[6] NA F, TA C (2003) Pre-mRNA splicing and human disease. Genes Dev 17: 419–437. 12600935 10.1101/gad.1048803

[7] Venables JP, Klinck R, Koh C, Gervais-Bird J, Bramard A, et al (2009) Cancer-associated regulation of alternative splicing. Nature structural & molecular biology 16: 670–676. 10.1038/nsmb.1608 19448617

[8] He C, Zuo Z, Chen H, Zhang L, Zhou F, et al (2007) Genome-wide detection of testis- and testicular cancer-specific alternative splicing. Carcinogenesis 28: 2484–2490. 17724370 10.1093/carcin/bgm194

[9] Gardina PJ, Clark TA, Shimada B, Staples MK, Yang Q, et al (2006) Alternative splicing and differential gene expression in colon cancer detected by a whole genome exon array. BMC genomics 7: 325. 17192196 10.1186/1471-2164-7-325 PMC1769375

[10] Ohnuma S, Miura K, Horii A, Fujibuchi W, Kaneko N, et al (2009) Cancer-associated splicing variants of the CDCA1 and MSMB genes expressed in cancer cell lines and surgically resected gastric cancer tissues. Surgery 145: 57–68. 19081476 10.1016/j.surg.2008.08.010

[11] David CJ, Manley JL (2010) Alternative pre-mRNA splicing regulation in cancer: pathways and programs unhinged. Genes & development 24: 2343–2364. 21041405 10.1101/gad.1973010 PMC2964746

[12] Seliger B (2008) Different regulation of MHC class I antigen processing components in human tumors. Journal of immunotoxicology 5: 361–367. 19404870 10.1080/15476910802482870

[13] Romero JM, Jiménez P, Cabrera T, Cózar JM, Pedrinaci S, et al (2005) Coordinated downregulation of the antigen presentation machinery and HLA class I/beta2-microglobulin complex is responsible for HLA-ABC loss in bladder cancer. International journal of cancer Journal international du cancer 113: 605–610. 15455355 10.1002/ijc.20499

[14] Norell H, Carlsten M, Ohlum T, Malmberg K-J, Masucci G, et al (2006) Frequent loss of HLA-A2 expression in metastasizing ovarian carcinomas associated with genomic haplotype loss and HLA-A2-restricted HER-2/neu-specific immunity. Cancer research 66: 6387–6394. 16778217 10.1158/0008-5472.CAN-06-0029

[15] Miranda NFCCD, Nielsen M, Pereira D, Puijenbroek MV, Vasen HF, et al.. (2009) MUTYH-associated polyposis carcinomas frequently lose HLA class I expression – a common event amongst DNA-repair-de cient colorectal cancers. Journal of Pathology, The: 69–76. doi:10.1002/path. 10.1002/path.2569 19462419

[16] Ferris RL, Whiteside TL, Ferrone S (2006) Immune escape associated with functional defects in antigen-processing machinery in head and neck cancer. Clinical cancer research: an official journal of the American Association for Cancer Research 12: 3890–3895. 16818683 10.1158/1078-0432.CCR-05-2750

[17] Watson NFS, Ramage JM, Madjd Z, Spendlove I, Ellis IO, et al (2006) Immunosurveillance is active in colorectal cancer as downregulation but not complete loss of MHC class I expression correlates with a poor prognosis. International journal of cancer Journal international du cancer 118: 6–10. 16003753 10.1002/ijc.21303

[18] Ge B, Gurd S, Gaudin T, Dore C, Lepage P, et al (2005) Survey of allelic expression using EST mining. Genome research 15: 1584–1591. 16251468 10.1101/gr.4023805 PMC1310646

[19] Sayers EW, Barrett T, Benson DA, Bolton E, Bryant SH, et al (2010) Database resources of the National Center for Biotechnology Information. Nucleic acids research 39: D38–D51. 21097890 10.1093/nar/gkq1172 PMC3013733

[20] Fang F, He X, Deng H, Chen Q, Lu J, et al (2007) Discrimination of metabolic profiles of pancreatic cancer from chronic pancreatitis by high-resolution magic angle spinning 1H nuclear magnetic resonance and principal components analysis. Cancer science 98: 1678–1682. 17727683 10.1111/j.1349-7006.2007.00589.x PMC11158482

[21] Urayama S, Zou W, Brooks K, Tolstikov V (2010) Comprehensive mass spectrometry based metabolic profiling of blood plasma reveals potent discriminatory classifiers of pancreatic cancer. Rapid communications in mass spectrometry: RCM 24: 613–620. 20143319 10.1002/rcm.4420

[22] Figge M A, Blankenship L (2004) Missense mutations in the BRCT domain of BRCA-1 from high-risk women frequently perturb strongly hydrophobic amino acids conserved among mammals. Cancer epidemiology, biomarkers & prevention: a publication of the American Association for Cancer Research, cosponsored by the American Society of Preventive Oncology 13: 1037–41. 15184261

[23] Kauzmann W (1959) Some factors in the interpretation of protein denaturation. Advances in protein chemistry 14: 1–63. 14404936 10.1016/s0065-3233(08)60608-7

[24] Figge MA, Blankenship L (2004) Missense mutations in the BRCT domain of BRCA-1 from high-risk women frequently perturb strongly hydrophobic amino acids conserved among mammals. Cancer epidemiology, biomarkers & prevention: a publication of the American Association for Cancer Research, cosponsored by the American Society of Preventive Oncology 13: 1037–1041. 15184261

[25] Wiedenfeld EA, Fernandez-Viña M, Berzofsky JA, Carbone DP (1994) Evidence for selection against human lung cancers bearing p53 missense mutations which occur within the HLA A*0201 peptide consensus motif. Cancer research 54: 1175–7. 8118802

[26] Koscielny G, Le Texier V, Gopalakrishnan C, Kumanduri V, Riethoven J-J, et al (2009) ASTD: The Alternative Splicing and Transcript Diversity database. Genomics 93: 213–220. 19059335 10.1016/j.ygeno.2008.11.003

[27] Kelso J, Visagie J, Theiler G, Christoffels A, Bardien S, et al (2003) eVOC: a controlled vocabulary for unifying gene expression data. Genome research 13: 1222–1230. 12799354 10.1101/gr.985203 PMC403650

[28] Wernersson R (2006) Virtual Ribosome–a comprehensive DNA translation tool with support for integration of sequence feature annotation. Nucleic acids research 34: W385–W388. 16845033 10.1093/nar/gkl252 PMC1538826

[29] Hoof I, Peters B, Sidney J, Pedersen LE, Sette A, et al (2009) NetMHCpan, a method for MHC class I binding prediction beyond humans. Immunogenetics 61: 1–13 Available: http://www.ncbi.nlm.nih.gov/pubmed/19002680. 19002680 10.1007/s00251-008-0341-z PMC3319061

[30] Nielsen M, Lundegaard C, Blicher T, Lamberth K, Harndahl M, et al (2007) NetMHCpan, a Method for Quantitative Predictions of Peptide Binding to Any HLA-A and -B Locus Protein of Known Sequence. PLoS ONE 2: e796. 17726526 10.1371/journal.pone.0000796 PMC1949492

[31] Lund O, Nielsen M, Kesmir C, Petersen AG, Lundegaard C, et al (2004) Definition of supertypes for HLA molecules using clustering of specificity matrices. Immunogenetics 55: 797–810. 14963618 10.1007/s00251-004-0647-4

[32] Zhang H, Lundegaard C, Nielsen M (2009) Pan-specific MHC class I predictors: a benchmark of HLA class I pan-specific prediction methods. Bioinformatics (Oxford, England) 25: 83–89. 10.1093/bioinformatics/btn579 PMC2638932 18996943

[33] Erup Larsen M, Kloverpris H, Stryhn A, Koofhethile CK, Sims S, et al (2011) HLArestrictor – a tool for patient-specific predictions of HLA restriction elements and optimal epitopes within peptides. Immunogenetics 63: 43–55. 21079948 10.1007/s00251-010-0493-5

[34] Rao X, Costa AICAF, van Baarle D, Kesmir C (2009) A comparative study of HLA binding affinity and ligand diversity: implications for generating immunodominant CD8+ T cell responses. Journal of immunology (Baltimore, Md: 1950) 182: 1526–1532. 10.4049/jimmunol.182.3.1526 19155500

[35] Hopp TP, Woods KR (1981) Prediction of protein antigenic determinants from amino acid sequences. Proceedings of the National Academy of Sciences of the United States of America 78: 3824–3828. 6167991 10.1073/pnas.78.6.3824 PMC319665

[36] Wimley WC, White SH (1996) Experimentally determined hydrophobicity scale for proteins at membrane interfaces. Nature structural biology 3: 842–848. 8836100 10.1038/nsb1096-842

[37] Simpson RJ (2002) Proteins and Proteomics: A Laboratory Manual. Cold Spring Harbor Laboratory Press. Available: http://www.amazon.com/Proteins-Proteomics-Laboratory-Richard-Simpson/dp/0879695544. Accessed 2011 Jul 18.

[38] Trinquier G, Sanejouand YH (1998) Which effective property of amino acids is best preserved by the genetic code? Protein engineering 11: 153–169. 9613840 10.1093/protein/11.3.153

[39] Richards FM (1977) Areas, volumes, packing and protein structure. Annual review of biophysics and bioengineering 6: 151–176. 10.1146/annurev.bb.06.060177.001055 326146

[40] Baumann G, Frömmel C, Sander C (1989) Polarity as a criterion in protein design. Protein engineering 2: 329–334. 2928295 10.1093/protein/2.5.329

[41] Darby NJ, Creighton TE (1993) Protein structure.

[42] Miller S, Janin J, Lesk AM, Chothia C (1987) Interior and surface of monomeric proteins. Journal of molecular biology 196: 641–656. 3681970 10.1016/0022-2836(87)90038-6

[43] Nielsen M, Lundegaard C, Worning P, Hvid CS, Lamberth K, et al (2004) Improved prediction of MHC class I and class II epitopes using a novel Gibbs sampling approach. Bioinformatics (Oxford, England) 20: 1388–1397. 10.1093/bioinformatics/bth100 14962912

[44] Schneider TD, Stephens RM (1990) Sequence logos: a new way to display consensus sequences. Nucleic acids research 18: 6097–6100. 2172928 10.1093/nar/18.20.6097 PMC332411

[45] Wilson E (1927) Probable Inference, the Law of Succession, and Statistical Inference. Journal of the American Statistical Association 22: 209–212.

